# Social sciences research in the Central European city of Wrocław: A density-equalizing mapping analysis

**DOI:** 10.1371/journal.pone.0205094

**Published:** 2018-10-24

**Authors:** David A. Groneberg

**Affiliations:** 1 The Institute of Occupational Medicine, Social Medicine and Environmental Medicine, Goethe-University, Frankfurt, Germany; KU Leuven, BELGIUM

## Abstract

**Background:**

The city of Wrocław in Poland represents one of Central Europeans oldest capitals of science with numerous Nobel laureates. Due to a long history of political suppressions with Nazi Germany and Communism from 1933 until 1989, its scientific community was suppressed for more than half a century.

**Methods:**

The present study assessed scientific activities in the field of social and neighbouring sciences using density equalizing mapping. On the basis of the NewQIS (New Quality and Quantity Indices in Science) platform and the Social Sciences Citation Index (SSCI) of the Web of Science database, a total of 1787 articles originating from Wrocław were identified between 1966 and 2017.

**Results:**

In total, 549 research collaborations of Wrocław with 96 different countries were present (30.7%). Among the 107 research areas the highest activity was found for the field of Business and Economics with n = 272 articles (average citation rate (AVR) of 12.54), followed by Psychology (n = 252 articles, AVR = 9.06), Psychiatry (n = 205 articles, AVR = 4.74) and Public, Environmental and Occupational Health (n = 145 articles, AVR = 7.96). The highest AVR was found for Operations Research (25.36 with n = 87 articles). Density equalizing mapping procedures revealed a global pattern of social sciences research collaborations with scientists from Germany, the UK and the US as the primary cooperating partner of Wrocław. The different countries had major differences in the area of research collaborations.

**Conclusions:**

This is the first study that depicts the global network of Wrocław scientific activities in the field of social sciences. The exorbitant increase in research activity from 2006 onwards can lead to the assumption that Wrocław social sciences encounter a fruitful future.

## 1. Introduction

The Polish city of Wrocław belongs to a number of central European cities which are famous for their long tradition in science. Being originally founded in 1702 its university constitutes the academic centre of Silesia since more than three centuries. The multi-national tradition of the 19th century with Polish, German, and Jewish students was destroyed during the era of Nazi Germany with more than 250 doctorate degrees being stripped by the Nazis [1–5]. After the second world war, Polish scientists opened a new academic life in Wrocław [1–5] and a number of separate universities were created: the University of Wrocław, the Wrocław University of Science and Technology, the Wrocław Medical University or the Wrocław University of Environmental and Life Sciences [1–5].

A number of historical articles and reviews already addressed the achievements made by Wrocław scientists in different areas of life sciences [6, 7]. However, there are also citations in the literature claiming that the "Bright light of learning snuffed out" in Wrocław after the Second World War [8]. In the light of these reports, the New Quality and Quantity in Sciences (NewQIS) platform initiated a new NewQIS-Wrocław project that addresses different areas of sciences in Wrocław including chemistry and biomedical sciences [9, 10]. NewQIS has been established in 2008/9 and was discussed as a valuable tool to analyse various aspects of scientific advancement in the past decade [11, 12]. The aim of the project is to elucidate scientific advancement in Wrocław by the use of novel visualization techniques and advanced scientometric tools [13, 14]. The present study specifically addressed social sciences and related areas.

## 2. Methods

### 2.1 NewQIS protocol

This study is embedded in the NewQIS (*New Quantity and Quality indices in Science*) platform which was established in 2009 [13, 14]. NewQIS is an international project encompassing currently more than 50 different scientometric studies in all areas of science. It ranges from the assessment of infectious diseases [15–17], tropical diseases [18], neglected diseases [19] to cancer [20–22], gynaecological issues [23, 24], medical technologies [25], accidents [26], to public health [27] and policy issues [28] or journals [29]. The present approach uses visualization algorithms including density equalizing mapping by Gastner and Newman [30], bibliometric indices such as the Hirsch-index [31]. Thus, the quantity assessment and quality estimation of a large data bases is enabled by the utilization of various indices. This provides important information that is necessary for researchers, planers, funders and decision makers.

### 2.2 Data source

In order to assess aspects of social sciences and related fields, the Social Sciences Citation Index (SSCI) of the Web of Science Core Collection was used. To identify all publication types related to Wrocław institutions, the search term *Wrocław* was entered in the address field.

### 2.3 Density-equalizing mapping

As previously described in numerous NewQIS articles, density-equalizing mapping was used to illustrate global aspects [32, 33]. Briefly, the national territories were re-sized according to the chosen variable, i.e. the number of published social sciences articles or citations related to the Wrocław articles [34, 35]. The precise mathematical calculations to re-size the countries are based on Gastner and Newman’s algorithm [30]. This allows a geographical assessment of a substantial amount of data in a short period of time.

### 2.4 Analysis of research of international collaborations

In order to assess the international networking processes of Wrocław social sciences research collaborations a matrix was constructed. This was performed as previously published [36, 37]. All networking countries related to Wrocław were visualized by the use of vector techniques. Bilateral collaborations between Wrocław and foreign country or between foreign countries were defined when at least one author originated from Wrocław and at least one other author from a Non-Polish affiliation [38, 39].

## 3. Results

### 3.1 Total research activity in social sciences

**Fig 1:**
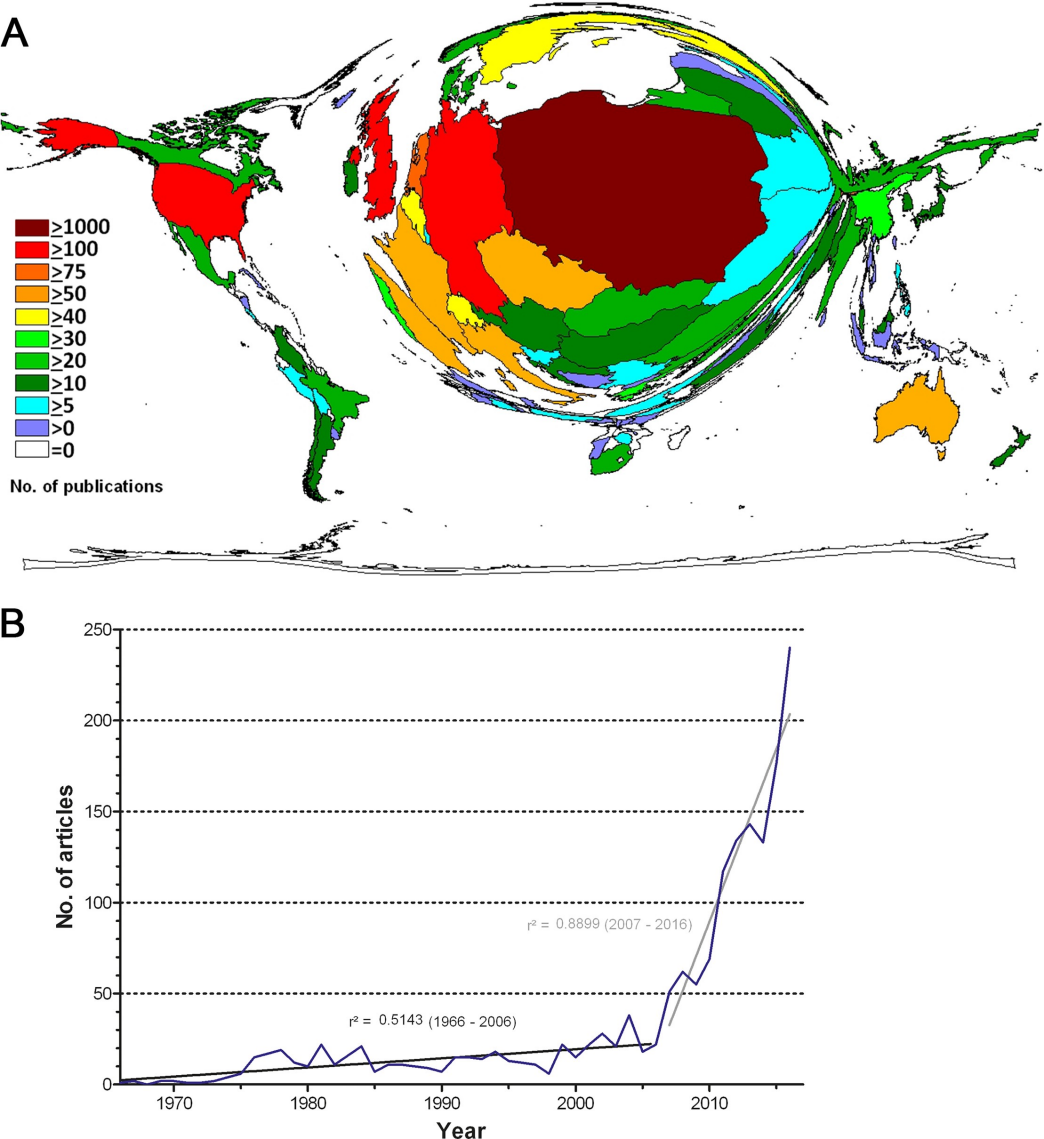
Number of publications worldwide. (A) Density equalizing map of the number of publications per country. B) Number of publications per year, r^2^ = correlation coefficient.

In total, a number of 1787 publications was identified in Social Sciences Citation Index (SSCI) of the Web of Science database being published by Wrocław scientists in the period from 1966 to 2017. As illustrated in Fig 1, less than 50 articles were published annually until 2006. Regression analysis demonstrates that there are two periods of research development that can be seen differently: The coefficient of determination over the whole evaluation period is with r^2^ = 0.49 comparatively low, whereas the period from 2007 to 2016 evaluated separately shows a strong relation (r^2^ = 0.89). The coefficient of the period from 1966 and 2006 with r^2^ = 0.51 is laying between. All correlations are highly significant (p<0,0001***, Spearman). Hence, a strong, homogenous increase in social sciences research activity in the past recent years can be stated, with the year 2016 holding the largest number of published items with nearly n = 250 (Fig 1B). To depict a world map of social sciences research related to Wrocław, density-equalizing mapping was applied. In a first step with the inclusion of the Wrocław, the resulting density equalizing map of the world is dominated by Poland (Fig 1A). The average citation rate (AVR) of the 1787 articles was 6.71 and the Hirsch (H) index of these articles was 46.

### 3.2 Global social sciences research network

**Fig 2:**
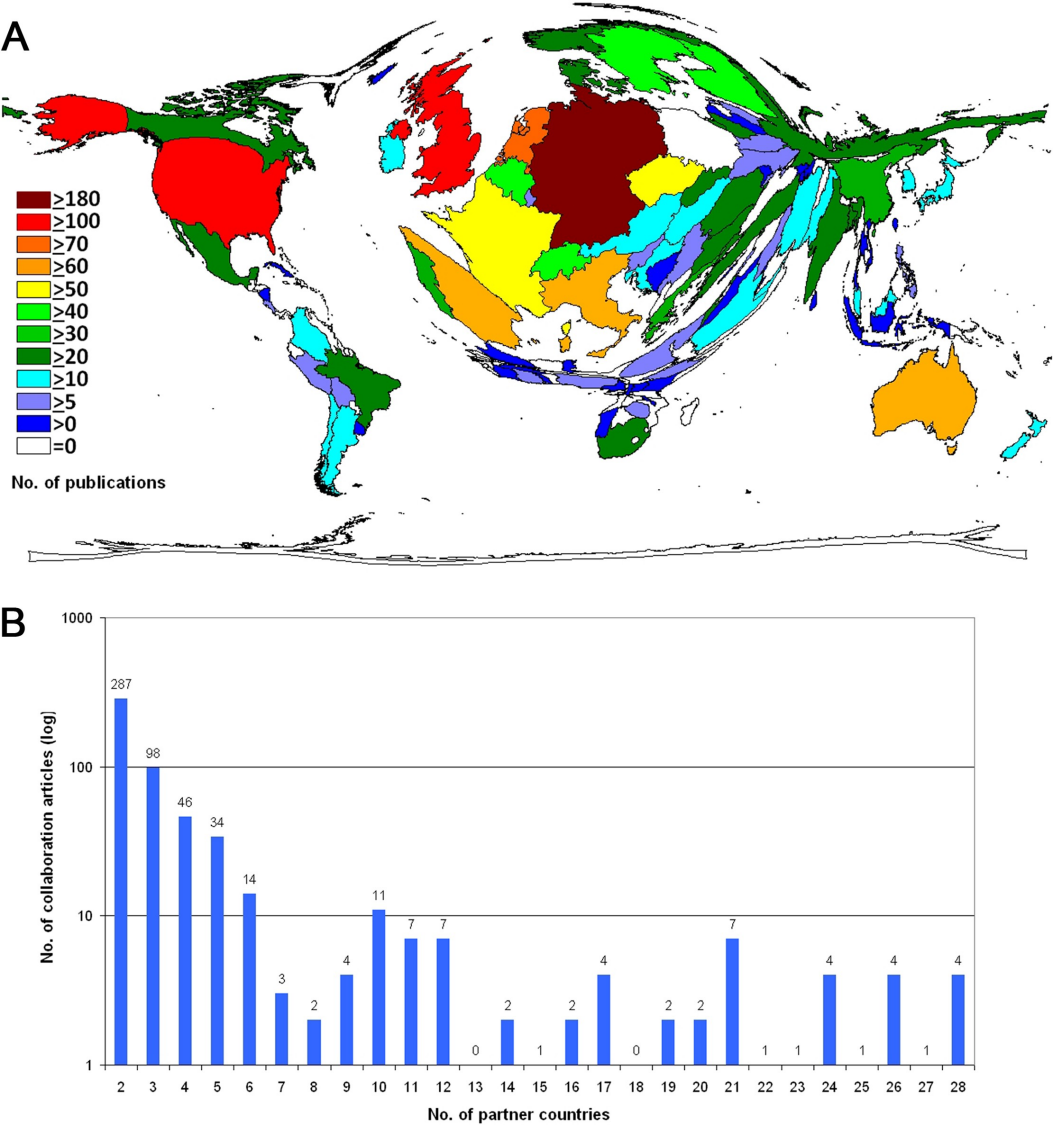
Number of publications of the collaborating countries. (A) Density equalizing map of the number of publications per country. (B) Number of collaborating partner countries per article and amount of articles.

**Fig 3:**
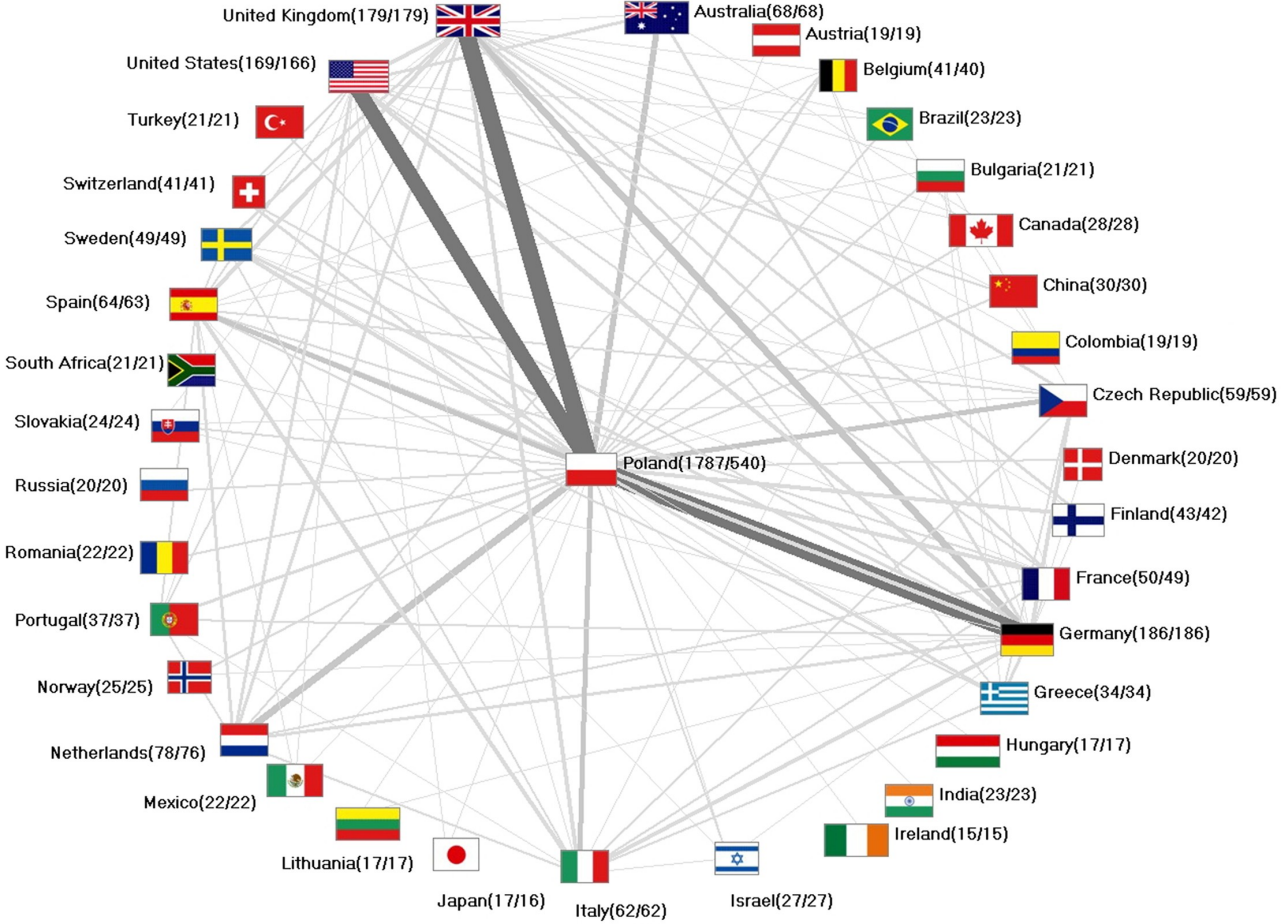
International network. Network diagram of the international collaboration partners, numbers in brackets (publications/collaboration articles).

**Fig 4:**
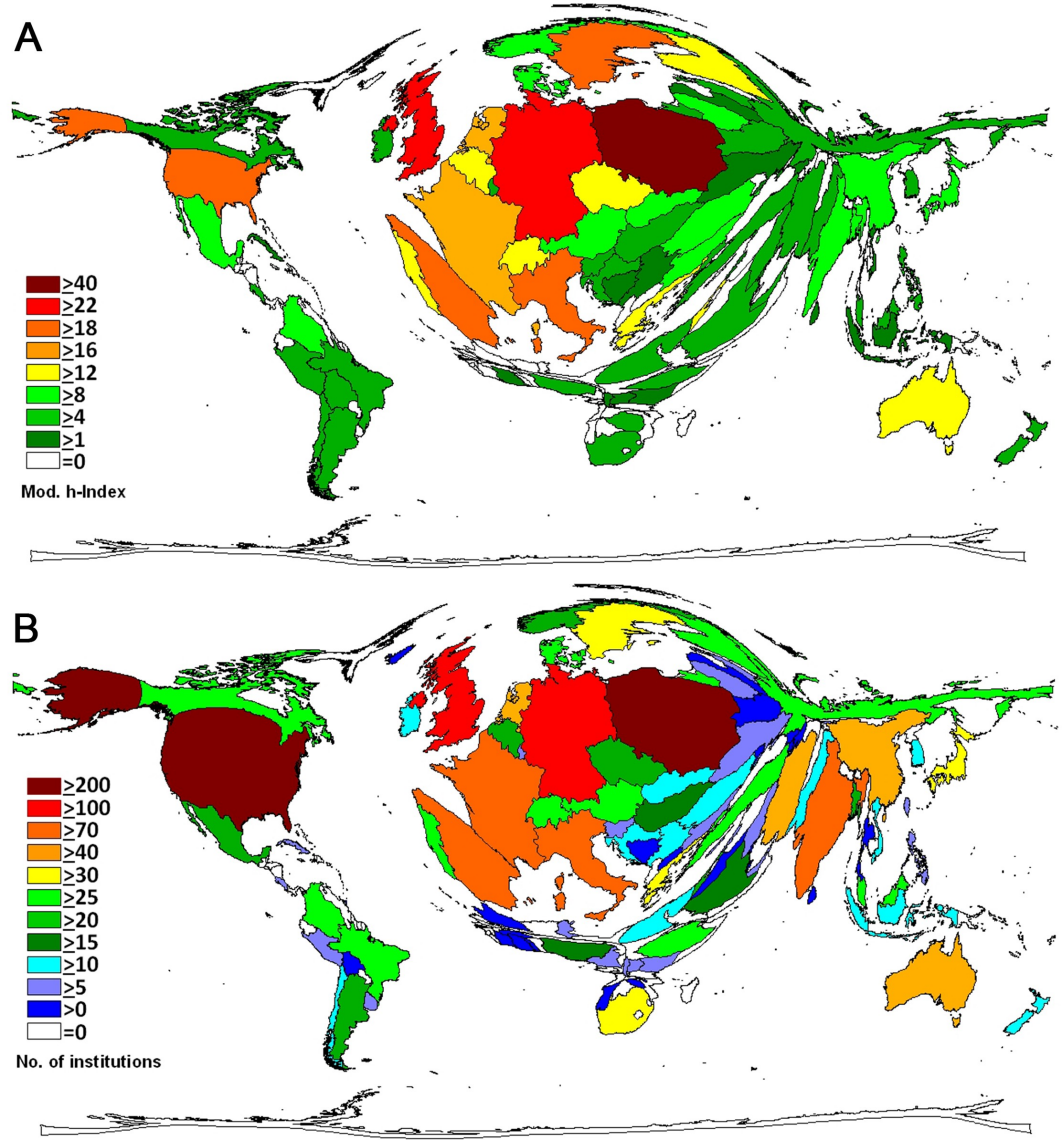
Density equalizing mapping (A) Modified h-indices of Wrocław and all other collaborating countries. (B) Number of collaborating institutions of Wrocław social sciences research in Poland and all collaborating countries.

A total of 549 social sciences research cooperations with 96 countries was conducted by Wrocław scientists. This is a percentage of 30.7% of all social sciences publications originating from Wrocław. Density-equalizing mapping was used to illustrate the global proportions of Wrocław social sciences research partner countries and the generated world map was distorted towards Germany (n = 186 joint articles), the UK (n = 178), the US (n = 169). Also, the Netherlands (n = 78), Australia (n = 68), Spain (n = 64), Italy (n = 62), Czech Republic (n = 59) and France (n = 50) had at least 50 collaborations with Wrocław scientists. (Fig 2A). Out of these 549 collaborations 287 were bilateral between Wrocław and one other country, n = 98 were trilateral and n = 46 were between Wrocław and scientists from three other countries. The highest number of countries collaborating in the field of social sciences was 28 (Fig 2B). Chart techniques were used (Fig 3) to visualize the primary collaborative partners of Wrocław and the network between these partners. The H index was used and modified as a semi-qualitative citation indicator to assess qualitative aspects of collaborative research. Density equalizing mapping shows that there are at least 22 collaborative articles with the UK and Germany that received at least 22 citations. Also, collaborative articles of Wrocław scientists with the US, Sweden, Spain and Italy displayed in H index of at least 18 (Fig 4A). Analysis of the affiliated institutions revealed 234 Polish institutions. 248 US institutions, 129 UK institutions and 105 German institutions were active in collaborations with Wrocław, followed by Italy (n = 92), France (n = 89) and Spain (n = 83). India was present with 73 institutions, China with n = 53 institutions, and Australia with n = 47 institutions (Fig 4B).

### 3.3 Research areas

**Fig 5:**
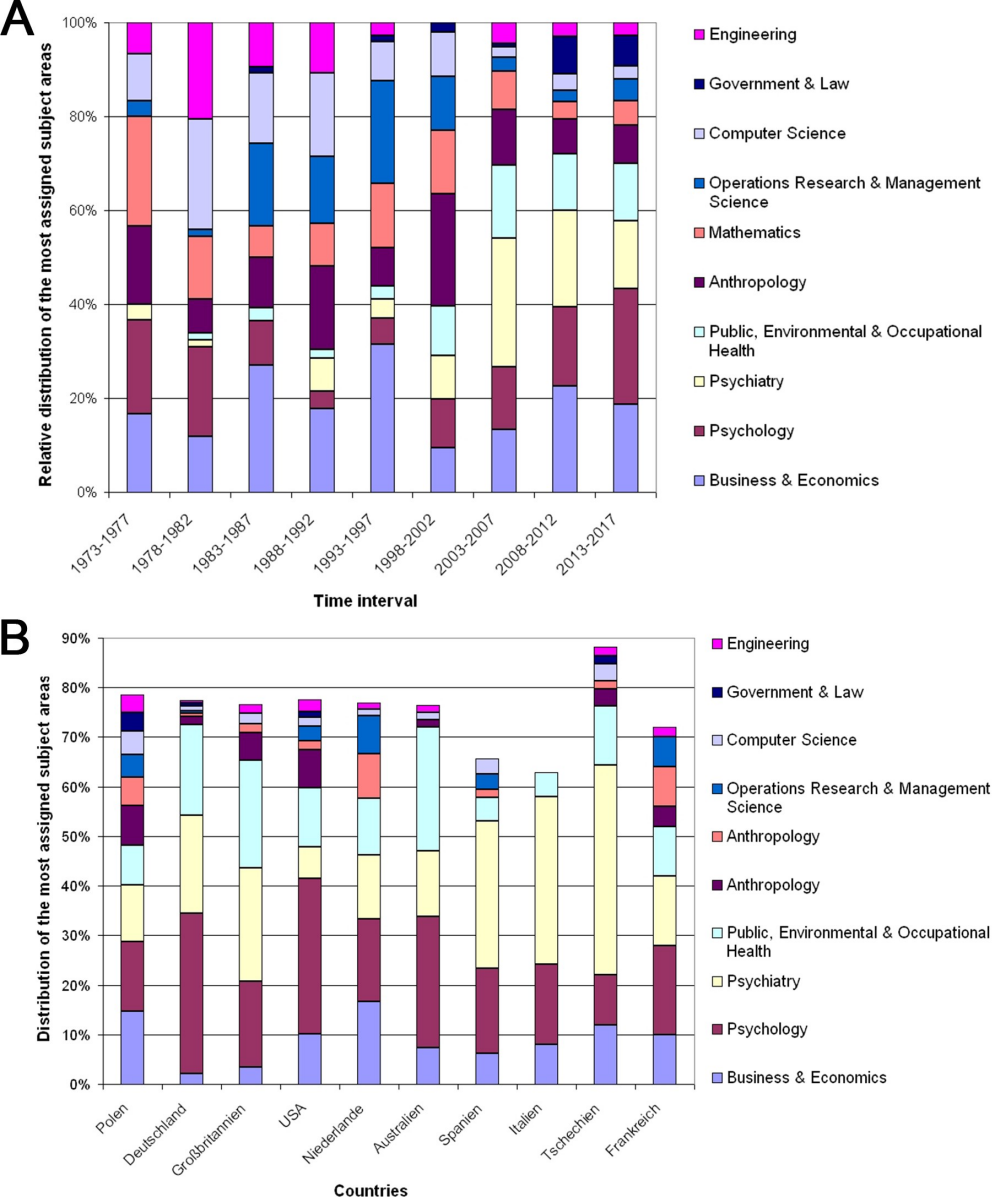
Subject areas with social sciences and related fields research. (A) Proportion of subjected areas in 5-year time intervals. (B) Proportion of subjected areas in the collaboration with other countries.

A total of 107 research areas are present in the 1787 Wrocław articles that were published since 1966. The most active field in the social sciences citation index was Business & Economics with n = 272 articles that were cited 3412 times. The next field was Psychology with N = 252 articles (cited 2284 times) and Psychiatry (n = 205 articles with 971 citations). Public, Environmental and Occupational Health aspects were present in n = 145 articles and cited 1154 times. They were followed by Anthropology (n = 144 articles, cited 678 times), Mathematics (n = 102 articles, cited 794 times). The highest citation rate was present in the field of Operations Research: n = 87 articles were cited 2206 times which is an average citation rate of 25.36. An analysis of the time evolution shows a dynamic picture with varying proportions of single fields between 1973 and 2002. Since 2003, the proportions consolidate. Now, the prominent fields are Psychology, Business and Economics, Psychiatry and Public, Environmental and Occupational Health (Fig 5A). Large variations were also present when the leading collaborative countries of Wrocław social sciences research were analysed: Wrocław-German, Wrocław- US and Wrocław- Australian collaborations were dominated by the field of Psychology. By contrast, Wrocław-UK collaborations had a high proportion of Public, Environmental and Occupational Health aspects. Collaborations with Spain, Italy and the Czech Republic were predominantly characterized by collaborations with aspects of Psychiatry (Fig 5B).

## 4. Discussion

In 2001, *Nature* published a correspondence of Min-Liang Wong, National Chung-Hsing University, Taiwan that addressed the Millennium Essay "Genius loci" of Vaclav Smil, University of Manitoba, Canada [8, 40]. This correspondence stated that Smil emphasized the importance of Budapest in twentieth-century science. With regard to Budapest, Wong proposed Wrocław as another locus in central Europe with the following renowned scientists and Nobel laureates: Max Born (Nobel Prize in Physics, 1954), Richard Courant (mathematician, 1888–1972), Paul Ehrlich (Nobel Prize in Physiology or Medicine, 1908), Fritz Haber (Nobel Prize in Chemistry, 1918), Reinhard Selten (Nobel Prize in Economic Sciences, 1994), Otto Stern (Nobel Prize in Physics, 1943) and Otto Toeplitz (mathematician, 1881–1940) [8]. The conclusion of the correspondence was that the scientific glory of the city was lost by the year 2001. Here, the field of social sciences and adjacent areas were analysed for scientific productivity, bearing in mind the difficult times that Wrocław academic institutions encountered during the terror of Nazi Germany and the communist era. With regard to the evolution of the scientific activity–measured in scholarly publication listed in the SSCI database of the Web of Science, the claim of Wong can be followed somehow until the beginning of the 21st century: There is only limited activity until 2006 present, as demonstrated by the low publication activities from 1966 onwards with less than 50 articles per year during the communist era and in the developing time afterwards. However, with the entry of Poland into the European Union there is a clear cut. The present data with a strong and homogenous (r^2^ = 0.8899) increase in scientific output from 2007 onwards clearly demonstrates that Wrocław scientists working in the fields of social sciences improved their publication output considerably. It can be discussed why the regain of scientific productivity started more than ten years after the end of the communist era in 2006–7:

Firstly, we found that in total, 772 Journals are listed in WoS that published SSCI indexed papers with participation of Wroclaw institutions until now. The strong increase of journals, that has been already discussed in former publications [41] [42] can be confirmed by the results of this study. Since 2000 (164 journals) the number of SSCI journals publishing articles of Wroclaw’s institutions rose to 308 journals in 2010 and nearly 5-fold until 2018. A study showed a similar development of all SSCI journals from 1998 (1679 journals) to 2009 (2,697 journals). [43] The number of Polish journals indexed for JCR (Journal Citation Report) is also growing steadily [44]. This indexing of Polish articles benefited from the inclusion of many journals from non-English speaking countries in WoS in 2005/2006. This can also be shown for the social sciences. Certainly, this the new indexing shows its influences in the increasing publication numbers of Wroclaw’s scientists, too. Looking at the SSCI, the first Polish journal has been indexed only since 2007. Until now, the number increased to a comparatively small number of 11 Polish journals. Additionally, the Journal Impact-Factor (JIF) and the indexing has been proofed to be significantly less important in social sciences [41]. This may change in future for Polish scientists. Since 2009 the regulations for research funding have been changed performance-based to a points-system that assesses the scientific institutions. JCR indexed Articles gain a high number of points. This contributed also to the increase of Wroclaw’s publication output.

Secondly, one theory is that the entry of Wrocław and Poland to the EU in 2004 and its funding programs gave a new boost to research activities. In this respect, Kozak et al. discussed [45] that the fall of the borders to the Western hemisphere did not only lead to a rapid benefit for the Eastern economies, but also improved scientific development. Especially collaborations with Western academic affiliations increased heavily. Hence, the landscape of Polish science changed dramatically in the last decade of the twentieth century[45]. However, from a total perspective of all Eastern European countries, the scientific development has not been as fruitful as in Wrocław. When Kozak et al. analysed the scientific output of scientists performance from Bulgaria, Belarus, the Czech Republic, Hungary, Moldova, Poland, Romania, Russia, Slovakia, and Ukraine from 1981 to 2011, it was found that the communist era breakdown did not generally lead to a huge increase in scientific activity. The authors concluded that most of the Eastern European countries were still subject to changes and are still awaiting their boost in scientific development. Hence, the present study illustrates that Wrocław scientists managed to gain back scientific strength in social sciences. With regard to the statement that the "Bright light of learning snuffed out in Breslau" after the Second World War [8] it can now be stated that the light is re-ignited again and coming generations of Wrocław scientists may encounter a fruitful academic atmosphere–if the political landscape allows this.

Next to the scientific activity in the SSCI which was presently assessed and visualized using density-equalizing maps [30], this study also sheds light on the evolutionary aspects of different fields of science in Wrocław that are registered in the Social Sciences Citation Index (SSCI): By the nature of the composition of the SSCI, it should be anticipated that fields close to medicine and life sciences such as the field of Psychiatry dominate the rankings. However, the analysis shows that among the 107 research areas which are present in the 1787 Wrocław articles–the most active field was Business & Economics with n = 272 articles. Since 2003, the proportional contributions are relatively similar with Psychology, Business and Economics, Psychiatry and Public, Environmental and Occupational Health being the leading areas concerning overall output. When it comes to citation of literature there are large differences present: Business and Economics have an average citation rate (AVR) of 12.54 citations per published article. This is higher than publications related to Psychology (n = 252 articles, AVR = 9.06), Psychiatry (n = 205 articles, AVR = 4.74) and Public, Environmental and Occupational Health (n = 145 articles, AVR = 7.96). The highest AVR was found for Operations Research with an AVR of 25.36 citations per article.

The present data also need to be interpreted with regard to the other NewQIS-Wrocław subprojects–biomedical research in Wrocław [10] and chemistry research in Wrocław [9]: The study on the biomedical research identified a total number of 10,366 articles in the Web of Science database being published in the period from 1972 to the end of 2016 [10]. For chemistry and related subjects, a total of 15,267 original research articles were found [9]. Since publication policies are different between these areas, i.e. in social sciences, scientists are more used to write monographs and book chapters than in biomedical sciences, the extent of these differences is not intriguing. More interesting is question if there are major differences concerning the international networking of social sciences versus chemistry and biomedical disciplines. This exemplifies that an important limitation of the current study is based upon the data base WoS with its sub-indices: Although biomedical and natural sciences are well represented in WoS, publications on humanities and social sciences are much less indexed in the subindex SSCI. Therefore, scientometric analyses are only applicable in a limited way and it is necessary bearing in mind that the absolute numbers of publications are not comparable to other disciplines and sub-indices such as the SCI [46]. As a matter of fact, the overwhelming majority of Polish social sciences publications is not indexed in the WoS. According to the Polish Scholarly Bibliography, in 2014 Polish scholars from the social sciences and humanities published 19,089 publications whereas according the WoS there are only 2,665 publications in 2014. Thus, only about 13.96% publications of Polish scholars are indexed in the WoS [47].

The percentage of collaborations is quite similar: 30.7% of publications in the field of social sciences, versus 28.% in biomedical and 28.6% in chemistry sciences were conducted within international collaborations [9, 10]. Interestingly, whereas the total number was lower for social sciences (549), there was a higher total number of collaborating countries with 96 for social sciences versus 83 countries for the field of chemistry (total number of collaboration articles = 4362). Only the field of biomedical sciences collaborated with more different countries (n = 104 different countries). A further important comparison is the country ranking of collaborations: For the field of social sciences, the ranking of most frequent collaborating countries was Germany (n = 186 joint articles), followed by the UK (n = 178), and the US (n = 169). The numbers were relatively similar (169 to 186 joint publications. Also, the Netherlands (n = 78), Australia (n = 68), Spain (n = 64), Italy (n = 62), Czech Republic (n = 59) and France (n = 50) had at least 50 collaborations with Wrocław scientists. For chemistry, the ranking was again very close: The US (n = 88), France (n = 658), Germany (n = 679) all were nearly at the same collaboration level. Interestingly, strong bonds were also present to Russia (n = 409) and Ukraine (n = 406), followed by Italy (n = 373) and the UK. By contrast, in the biomedical field, the ranking was Germany (n = 908), followed by the US (n = 784), the UK (n = 558). Also, Italy (n = 449) and France (n = 361) belong to the top five collaborating countries [9, 10]. The comparison of these rankings demonstrates that there are strong bonds to Germany as neighboring country and also to the US which represent the most active nation concerning research and development from a global perspective. The important role of France for Wrocław collaborations might reflect the generally strong bonds between Poland and France which is present since centuries.

However, the comparison of the present data to the previously published data on biomedical and chemical research in Wroclaw is also limited due to methodological issues: Although, the methodological approach and data sources are the same in the present study and the two previous studies, the subject of the study is different. Social sciences can not be easily compared to natural sciences because the publishing behavior of scientists is different between these fields of academia. Monographs are more common in social sciences than in natural or biomedical sciences. Since monographs are not commonly included to the SSCI, this large part of social sciences research activities is not included. Therefore, an overall comparison on absolute research activities should not be performed on the basis of the current data.

## 5. Conclusion

This is the first concise study that specifically addresses scientometric aspects of Wrocław social sciences activity in a period of more than fifty years, beginning in the communist era in 1966 and ending in the democratic era in 2017. It clearly shows that in contrast to reports focussing on other Eastern European countries [45], scientific life in Wrocław is active and rapidly grows since 2006/2007. This might be influenced by the entrance of Poland into the EU with all the different research funding programs and networking opportunities on the European level as well as the alteration of Polish research funding and the increasing opportunity for non-English countries to get listed in WoS. The importance of the awareness and reputation of the scientific performance is backed up by the necessity of global networking and the promotion of all research disciplines, especially for the social sciences all over the world. On the basis of the current analysis, detailed a compelling historical pieces may now focus on single sections of social sciences activities in Wroclaw over the past decades.
